# Effect of CYP3A4 Inhibitors and Inducers on Pharmacokinetics and Pharmacodynamics of Saxagliptin and Active Metabolite M2 in Humans Using Physiological-Based Pharmacokinetic Combined DPP-4 Occupancy

**DOI:** 10.3389/fphar.2021.746594

**Published:** 2021-10-19

**Authors:** Gang Li, Bowen Yi, Jingtong Liu, Xiaoquan Jiang, Fulu Pan, Wenning Yang, Haibo Liu, Yang Liu, Guopeng Wang

**Affiliations:** ^1^ Beijing Adamadle Biotech Co, Ltd., Beijing, China; ^2^ Xiyuan Hospital, China Academy of Chinese Medical Sciences, Beijing, China; ^3^ School of Chinese Materia Medica, Beijing University of Chinese Medicine, Beijing, China; ^4^ Chinese Academy of Medical Sciences and Peking Union Medical College, Institute of Medicinal Plant Development, Beijing, China; ^5^ Zhongcai Health (Beijing) Biological Technology Development Co, Ltd., Beijing, China

**Keywords:** saxagliptin, DDI prediction, DPP-4 occupancy, PBPK-DO model, CYP3A4

## Abstract

We aimed to develop a physiological-based pharmacokinetic and dipepidyl peptidase 4 (DPP-4) occupancy model (PBPK-DO) characterized by two simultaneous simulations to predict pharmacokinetic (PK) and pharmacodynamic changes of saxagliptin and metabolite M2 in humans when coadministered with CYP3A4 inhibitors or inducers. Ketoconazole, delavirdine, and rifampicin were selected as a CYP3A4 competitive inhibitor, a time-dependent inhibitor, and an inducer, respectively. Here, we have successfully simulated PK profiles and DPP-4 occupancy profiles of saxagliptin in humans using the PBPK-DO model. Additionally, under the circumstance of actually measured values, predicted results were good and in line with observations, and all fold errors were below 2. The prediction results demonstrated that the oral dose of saxagliptin should be reduced to 2.5 mg when coadministrated with ketoconazole. The predictions also showed that although PK profiles of saxagliptin showed significant changes with delavirdine (AUC 1.5-fold increase) or rifampicin (AUC: a decrease to 0.19-fold) compared to those without inhibitors or inducers, occupancies of DPP-4 by saxagliptin were nearly unchanged, that is, the administration dose of saxagliptin need not adjust when there is coadministration with delavirdine or rifampicin.

## Introduction

Patients undergoing multiple comorbidities are usually treated by complicated polypharmacy schemes and long-term administration, which means that they will be at the risk of drug–drug interactions (DDIs). In DDIs, one drug may affect the PK or PD behavior of another drug, thereby resulting in a series of side effects and even a withdrawal of approved pharmaceuticals from the market, for instance, mibefradil and terfenadine (Valicherla et al., 2019; Pelkonen et al., 2020). Therefore, prospective assessment of a potential risk of DDIs is significant within the pharmaceutical industry, such as guaranteeing the safety and reducing unnecessary consumption of new drugs. Cytochrome P450, which is the most comprehensive metabolizing enzyme in the gut and the liver, plays an important role in pharmacokinetic (PK) interaction-related DDIs through the interfering metabolism of most drugs (Dmitriev et al., 2019). Currently, PK-related DDIs mediated by CYP have become a research focus over the past decades (Conner et al., 2018; Peng et al., 2021). However, only focusing on PK-related DDIs is far from enough, and pharmacodynamic (PD)-related DDIs should receive more attention, particularly on dose adjustment for patients. Although PD-related DDIs recorded are roughly 1.9-fold higher than PK-related DDIs (Spanakis et al., 2019), unfortunately, simulation study on PD-related DDIs is far less compared to those on PK-related DDIs.

Ketoconazole and delavirdine are strongly competitive and time-dependent inhibitors (TDIs) against the CYP3A4 enzyme, and rifampicin is a strong CYP3A4 inducer. It is well known that ketoconazole and rifampicin are often recommended to assess potential DDIs for other drugs mainly metabolized by the CYP3A4 enzyme (Rytkönen et al., 2020; Xu et al., 2021). To our knowledge, owing to irreversible loss of P450 enzyme functions, TDIs are regarded to have a longer persistent time on the drug metabolic enzyme and consequently to cause clinically more significant DDIs in contrast to competitive inhibitors (Kosaka et al., 2017; Eng et al., 2020; Tanna et al., 2021). Therefore, ketoconazole, delavirdine, and rifampicin were chosen for potential DDI assessments.

Saxagliptin is an orally potent competitive DPP-4 inhibitor and utilized for the treatment of type 2 diabetes at a daily dose of 5 mg.^1^. Saxagliptin is metabolized extensively in humans *via* CYP3A4 to be converted into many metabolites, of which 5-hydroxy saxagliptin (M2) is the most major active metabolite. M2 *in vivo* is roughly in a 2-fold higher amount (44.1 versus 24.0%) and binds to DPP-4 with a ∼2-fold lower affinity than saxagliptin (1.3 versus 2.6 nm)^1^ (Su et al., 2012). Hence, when coadministered with inhibitors or inducers of CYP3A4, significant effects of them on PK and PD of saxagliptin should be taken into account.

We aimed to develop a mathematical model to assess the dynamic effect of ketoconazole, delavirdine, and rifampicin on the PK and PD of saxagliptin tablets in humans, when coadministered. More precisely, the physiological-based pharmacokinetic (PBPK) model and the DPP-4 occupancy (DO) model were incorporated into a new mathematical model, termed as the PBPK-DO model, which enables the changes of PK and PD of saxagliptin and M2 in humans to be quantified simultaneously with coadministration of CYP3A4 inhibitors or inducers.

## Methods

### Data Collection

Clinical PK studies of ketoconazole, delavirdine, and rifampicin were taken from the published literature^2, 3^(Hanke et al., 2018), which could be used in their respective PBPK model establishment and verification. Clinical PK and PD studies of saxagliptin and metabolite M2 were collected from published data (Upreti et al., 2011), which could be used in the PBPK-DO model development and verification for saxagliptin and M2. Physicochemical properties of four drugs, binding kinetics of saxagliptin and M2, and physiological parameters in humans required in developing the PBPK-DO model were obtained from published scientific studies, and the built-in libraries of Gastroplus software were used in this study, including Berkeley Madonna (Version 10.2.8, Berkeley Madonna, Inc Albany, CA, United States) and ADMET Predictor (Version 9.0.0.0, Simulation Plus, Inc Lancaster, CA, United States).

### Development of the PBPK-DO Model

**FIGURE 1:**
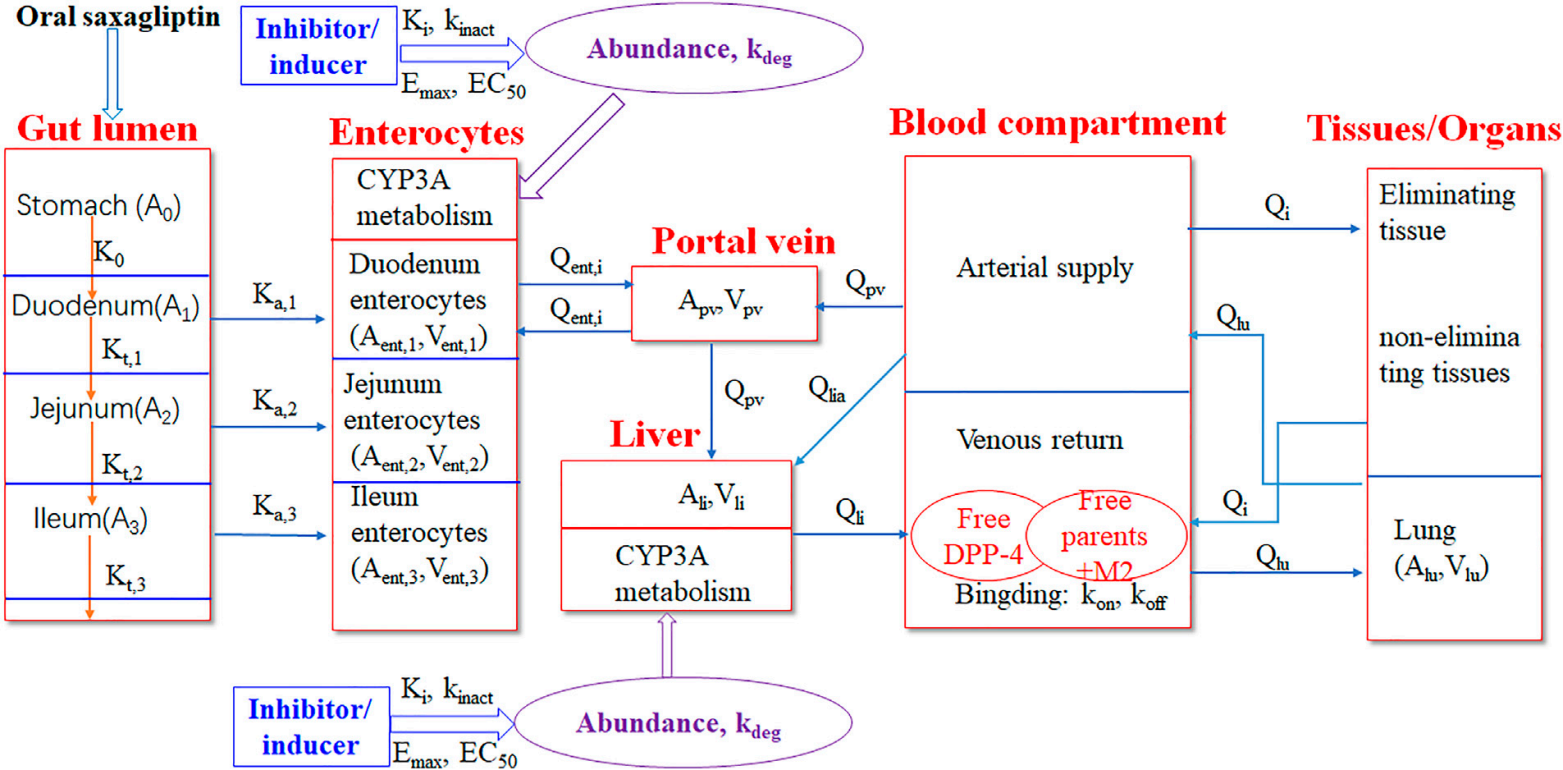
Schematic representation of the PBPK-DO model for saxagliptin in humans. The PBPK-DO model includes the gut lumen, enterocytes, the portal vein, the liver, blood (arterial supply and venous return), the lung, and other eliminating tissues (kidney) and non-eliminating tissues (11 compartments). The CYP3A metabolism enzyme was set into enterocytes and liver compartments. DPP-4 was assumed to only reside in the venous return compartment.

A PBPK-DO model was developed to simulate PK and DPP-4 occupancy time profiles of saxagliptin and metabolite M2 simultaneously after oral coadministration with ketoconazole, delavirdine, and rifampicin. The PBPK-DO model was composed of three key simulation processes, that is, PK prediction of saxagliptin and M2, simulation of DPP-4 occupancy by saxagliptin and M2, and interaction prediction with inhibitors/inducers of CYP3A4. In this PBPK-DO model, first, the simulation of saxagliptin and M2 concentration changes over time was enabled simultaneously by the PBPK model, which consisted of a stomach–gut compartment, a enterocytes compartment, a portal vein compartment, a blood compartment (arterial and venous blood), eliminating tissues (the liver, the kidney), non-eliminating tissues (adipose, the bone, the brain, the heart, muscle, the skin, and the spleen), and the lung. Next, the interactions between inhibitors/inducers and CYP3A4 were calculated through inhibition or inducing parameters (K_i_, K_inact_, E_max_, and EC_50_), CYP3A4 expression amount, and free saxagliptin concentration in the gut and liver. Eventually, time courses of DPP-4 occupancy were characterized by two key rate constants of on-rate (k_on_) and off-rate (k_off_) combined with free drug concentration around the DPP-4. In addition, we assumed that DPP-4 was located in the venous blood compartment in the present model. The overall framework of the PBPK-DO model is represented in Figure 1.

#### Stomach–Gut Compartment

Assuming that the drug in the stomach was neither absorbed nor metabolized, the drug amount in the stomach (A_0_) is only governed by the gastric emptying rate (K_0_). The change in mass within the stomach is described as follows (Qian et al., 2019):

$$\frac{dA_{0}}{dt}=-K_{0}\times A_{0}.$$ (1)

The gut lumen consists of the duodenum, jejunum, and ileum, and the amount of drug in the gut lumen (A_i_) is controlled by the gut transit rate constant (K_t,i_) and absorption rate constant (K_a,i_). The change in mass within each gut lumen was described as follows (Qian et al., 2019):

$$\frac{dA_{i}}{dt}=K_{t,i-1}\times A_{i-1}-K_{t,i}\times A_{i}-K_{a,i}\times A_{i},$$ (2)

where *i* = 1, 2, and 3 corresponds to the duodenum, jejunum, and ileum, respectively. The calculation of the K_a,i_ value is given by the following:^4^

$$K_{a,i}=K_{a,i0}\times A_{i}\times f_{a},$$ (3)

where K_a,i0_ represents the initial absorption rate constant and f_a_ represents the absorption adjustment factor. The calculation of the K_a,i0_ value is given by the following:^6^

$$K_{a,i0}=\frac{2\times P_{eff,human}\times ASF}{r_{i}},$$ (4)

where P_eff, human_ is the effective permeability in humans and r_i_ is the average radius of different intestinal segments (the duodenum, jejunum, and ileum). ASF is the absorption scale factor.

#### Enterocyte Compartment

Each different intestinal segment is linked with its enterocytes compartment. The amount of drug in each enterocyte compartment (A_ent,i_) was calculated according to the following equations (Qian et al., 2019):

$$\frac{dA_{ent,i}}{dt}=K_{a,i}\times A_{i}+Q_{ent,i}\times \frac{A_{pv}}{V_{pv}}-\left(Q_{ent,i}+f_{ugut}\times CL_{int,ent}\left(t\right)\right)\times \frac{A_{ent,i}}{V_{ent,i}\times K_{p,ent}},$$ (5)

$${\text{CL}}_{int,ent,i}\left(\text{t}\right)=\frac{V_{max}\times Abundance_{ent,i}\left(t\right)\times ISEF}{K_{m}+f_{ugut}\times \frac{A_{ent,i}\left(t\right)}{V_{ent,i}\times K_{p,ent}\times M}}\;,$$ (6)

where Q_ent,i_ and V_ent,i_ represent the blood flow rate and the volume of each enterocyte compartment, respectively. V_pv_ is the volume of the portal vein compartment, f_ugut_ is the free drug concentration in the enterocyte compartment, and K_p,ent_ is the ratio of intestine over blood drug concentration.

CL_int,ent_ (t) and Abundance_ent,i_ (t) represent the intrinsic metabolic clearance and CYP3A content in the enterocyte compartment with inhibitors/inducers or without inhibitors/inducers, respectively. V_max_ and K_m_ are metabolic parameters of the drug in each different enterocyte compartment. According to the reported result, saxagliptin is a weak P-glycoprotein substrate (Boulton, 2017); consequently, the efflux effect of P-glycoprotein on the amount of saxaliptin in the gut lumen was not considered in this study.

#### Portal Vein Compartment

The drug from the enterocyte compartment enters the liver *via* the portal vein, and hence, the change of drug amount over time in the portal vein compartment (A_pv_) is illustrated according to the following equations (Li et al., 2012; Qian et al., 2019):

$$\frac{dA_{pv}}{dt}=Q_{pv}\times C_{ab}+\overset{3}{\underset{i=1}{\sum }}Q_{ent,i}\times \frac{A_{ent,i}}{V_{ent,i}\times K_{p,ent}}-\overset{3}{\underset{i=1}{\sum }}Q_{ent,i}\times \frac{A_{pv}}{V_{pv}}-Q_{pv}\times \frac{A_{pv}}{V_{pv}},$$ (7)

where C_ab_ is the drug concentration in arterial blood.

#### Liver Compartment

The liver is a main eliminating tissue of saxagliptin, and the amount of drug in the liver is described as follows^6^ (Li et al., 2012):

$$\frac{dA_{li}}{dt}=Q_{pv}\times \frac{A_{pv}}{V_{pv}}+Q_{lia}\times C_{ab}-Q_{li}\times \frac{A_{li}\times Rbp}{V_{li}\times K_{p,li}}-{\text{CL}}_{int,li}\left(t\right)\times \frac{A_{li}\times f_{up}}{V_{li}\times K_{p,li}},$$ (8)

$$CL_{int,li}\left(t\right)=\frac{V_{max}\times Abundance_{li}\left(t\right)\times ISEF}{K_{m}+\frac{A_{li}\left(t\right)\times f_{up}}{V_{li}\times M\times K_{p,li}}}\;,$$ (9)

where A_li_, V_li_, Q_li_, and Q_lia_ are the drug amount in the liver, the volume and hepatic blood flow, and the hepatic artery blood flow rate of the liver, respectively. Rbp is the blood-to-plasma concentration ratio, f_up_ is the fraction of free drug in the plasma, and K_p,li_ is liver-to-plasma partition coefficient. M is molecular weight of the drug.

CL_int,li_(t) represents the intrinsic metabolic clearance of a drug in the liver. Abundance_li_(t) represents the hepatic CYP3A amount with inhibitors/inducers or without inhibitors/inducers. ISEF is the intersystem extrapolation factor. Owing to the finding that saxagliptin is not almost eliminated through biliary excretion (Fura et al., 2009), biliary clearance of saxagliptin is not incorporated into this PBPK model.

The amount of metabolite in the enterocyte compartment (A_met,ent_) is illustrated by the following:

$$\frac{dA_{met,ent}}{dt}=\overset{3}{\underset{i=1}{\sum }}\;\frac{f_{scale}\times V_{max}\times Abundance_{ent,i}\left(t\right)\times \frac{A_{ent,i}\left(t\right)\times f_{ugut}}{V_{ent,i}\times K_{p,ent}}}{K_{m}+\frac{A_{ent,i}\left(t\right)\times f_{ugut}}{V_{ent,i}\times M\times K_{p,ent}}}.$$ (10)

The amount of metabolite in the liver (A_met,li_) is illustrated by the following:

$$\frac{dA_{met,li}}{dt}=\frac{f_{scale}\times V_{max}\times Abundance_{li}\left(t\right)\times \frac{A_{li}\left(t\right)\times f_{up}}{V_{li}\times K_{p,li}}}{K_{m}+\frac{A_{li}\left(t\right)\times f_{up}}{V_{li}\times M\times K_{p,li}}},$$ (11)

where f_scale_ is the scale factor of metabolite conversion. The total amount of metabolite (A_met_) enters the venous blood compartment directly without consideration of gut absorption and is calculated by the following:

$$\frac{dA_{met}}{dt}=A_{met,ent}\left(t\right)+A_{met,li}\left(t\right)-K_{a,met}\times A_{met},$$ (12)

$$K_{a,met}=K_{a,met0}\times A_{met}\times f_{a,met},$$

where K_a,met0_ and K_a,met_ are the initial absorption rate constant and absorption rate constant of metabolite M2, respectively. f_a,met_ represents the absorption adjustment factor of the metabolite.

#### Kidney Compartment

The change of drug amount with time within the kidney (A_ki_) is described by the following (Li et al., 2012):

$$\frac{dA_{ki}}{dt}=Q_{ki}\times C_{ab}-Q_{ki}\times \frac{A_{ki}\times Rbp}{V_{ki}\times K_{p,ki}}-\frac{A_{ki}\times f_{up}\times CLr}{V_{ki}\times K_{p,ki}},$$ (14)

where Q_ki_ and V_ki_ are the blood flow and the volume of the kidney, respectively. K_p,ki_ is the kidney-to-plasma partition coefficient. CLr is the kidney elimination rate of the drug.

#### Lung Compartment

The amount of drug in the lung (A_lu_) is described as follows (Li et al., 2012):

$$\frac{dA_{lu}}{dt}=Q_{lu}\times C_{vb}-Q_{lu}\times \frac{A_{lu}\times Rbp}{V_{lu}\times K_{p,lu}},$$ (13)

where Q_lu_ and V_lu_ are the blood flow and the volume of the lung, respectively. K_p,lu_ is lung-to-plasma partition coefficient.

#### Other Non-eliminating Tissue Compartments

Overall, the amount of drug within non-eliminating tissue compartments (A_nt_) (the adipose, the bone, the brain, the heart, the muscle, the skin, the spleen, the red marrow, the yellow marrow, reproductive organs, and the rest of the body), except for the lung, follows the following equation (Li et al., 2012):

$$\frac{dA_{nt}}{dt}=Q_{nt}\times C_{ab}-Q_{nt}\times \frac{A_{nt}\times Rbp}{V_{nt}\times K_{p,nt}},$$ (16)

where Q_nt_ and V_nt_ are the blood flow sum and the mean volume of other non-eliminating tissues, respectively. K_p,nt_ is the partition coefficient sum of other non-eliminating over plasma.

#### Arterial Blood Compartment

The drug concentration within the arterial blood (C_ab_) is described as follows (Li et al., 2012; Fu et al., 2019):

$$\frac{dC_{ab}}{dt}=\frac{1}{V_{ab}}\times \left(Q_{lu}\times \frac{A_{lu}\times Rbp}{V_{lu}\times K_{p,lu}}-\underset{i}{\sum }Q_{i}\times C_{ab}\right),$$ (17)

where V_ab_ is the volume of the arterial blood compartment. *i* represents all eliminating and non-eliminating tissues except for the lung tissue.

#### Venous Blood Compartment

The drug concentration within the venous blood (C_vb_) is described as follows (Li et al., 2012; Fu et al., 2019):

$$\frac{dC_{vb}}{dt}=\frac{1}{V_{vb}}\times \left(\underset{i}{\sum }Q_{i}\times \frac{A_{i}\times Rbp}{V_{i}\times K_{p,i}}-Q_{lu}\times C_{vb}\right),$$ (18)

where V_vb_ is the volume of the venous blood compartment. *i* represents all eliminating and non-eliminating tissues except for the lung tissue.

#### CYP3A Dynamics of Inhibition and Induction

Here, the inhibition and induction model of CYP3A were developed assuming that intestinal inhibition and induction dynamic parameters were identical to hepatic dose. The CYP3A reversible competition inhibition dynamics is described as follows (Baneyx et al., 2014):

$$\frac{dAbundance_{i}}{dt}=\frac{-IN_{li}\times f_{up,in}\times Abundance_{i}}{K_{p,li,in}\times M_{in}\times \left(K_{i}+\frac{f_{up,in}\times IN_{li}}{K_{p,in}\times M_{in}}\right)}+k_{deg}\times \left(Abundance_{0}-Abundance_{i}\right),$$

The CYP3A time-dependent inhibition dynamics is described as follows (Baneyx et al., 2014):

$$\frac{dAbundance_{i}}{dt}=\frac{-k_{inact}\times I_{li}\times f_{up,in}\times Abundance_{i}}{K_{p,li,in}\times M_{in}\times \left(K_{i}+\frac{f_{up,in}\times I_{li}}{K_{p,in}\times M_{in}}\right)}+k_{deg}\times \left(Abundance_{0}-Abundance_{i}\right),$$ (20)

The CYP3A induction dynamics is described by the following (Qian et al., 2019):

$$\frac{dAbundance_{i}}{dt}=\frac{k_{deg}\times E_{max}\times I_{li}\times f_{up,in}\times Abundance_{i}}{K_{p,li,in}\times M_{in}\times \left(EC_{50}+\frac{f_{up,in}\times I_{li}}{K_{p,in}\times M_{in}}\right)}+k_{deg}\times \left(Abundance_{0}-Abundance_{i}\right)\;,\left(21\right)$$

where I_li_ is the concentration of the inhibitor/inducer within the targeted tissue (the gut and liver). f_up,in_ is the fraction of the free inhibitor/inducer in the plasma. K_p,in_ is the partition coefficient of the inhibitor/inducer of the targeted tissue (the gut and liver) to plasma. M_in_ is the molecular weight of the inhibitor/inducer. K_i_ and k_inact_ are inactivation parameters of the inhibitor against CYP3A4. EC_max_ and EC50 are inductive parameters of the inducer on CYP3A4. k_deg_ is the degradation rate constant of CYP3A4, and in this study, it was assumed that the k_deg_ value in the liver is identical to that in the gut.

#### DPP-4 Engagement Dynamics by Saxagliptin and Metabolite M2

The DPP-4 engagement time course by saxagliptin and metabolite M2 is described by a series of equations as follows (de Witte et al., 2016):

$$\frac{dTC}{dt}=\overset{2}{\underset{i=1}{\sum }}\left(k_{on,i}\frac{C_{vb,i}\times f_{up,i}}{M,i}-k_{off,i}\times TC,i\right),$$ (22)

$$\frac{dT_{free}}{dt}=\overset{2}{\underset{i=1}{\sum }}\left(-k_{on,i}\frac{C_{vb,i}\times f_{up,i}\times T_{free}}{M,i}+k_{off,i}\times TC,i\right),$$ (23)

$$T_{total}=T_{free}+\text{TC,}$$ (24)

$$\text{TO}=\overset{2}{\underset{i=1}{\sum }}\frac{TC_{i}}{T_{total}}\times 100\;,$$ (25)

where *i* = 1 and 2 corresponds to saxagliptin and active metabolite M2, respectively. TC is the concentration of saxagliptin/M2-DPP-4 complex formed. T_free_ is the concentration of free DPP-4. T_total_ is the sum of TC plus T_free_. C_vb,i_, k_on,i_, and k_off,i_ are the drug concentration within the venous blood and the on-rate and off-rate of saxagliptin and active metabolite M2, respectively.

### DDI Prediction Through the PBPK-DO Model

The DDI predictions were conducted to evaluate the parameter changes of PD (TO_AUC_, area under the occupancy–time curve; TO_max_, maximum occupancy; and DTO_>60%_ duration of >60% TO) and PK (AUC, area under the concentration–time curve; and C_max_, peak concentration) on saxagliptin and M2 with and without inhibitors/inducers.

## Results

### Data Collection of the PBPK-DO Model

The input parameters of saxagliptin and metabolite M2 for the PBPK-DO model are given in Table 1 (Rodgers et al., 2005; Kim et al., 2006; Rodgers and Rowland, 2006; Su et al., 2012; Qian et al., 2019). The corresponding parameters of inhibitors/inducers are listed in Supplementary Tables S1–S3 (Rodgers et al., 2005; Zhou et al., 2005; Rodgers and Rowland, 2006; Usach et al., 2013; Qian et al., 2019; Motiei et al., 2021; Lanni et al., 2021). The physiological parameters in humans are presented in Supplementary Table S4 (Li et al., 2012 and Qian et al., 2019)^6^.

**TABLE 1:** Summary of input parameters for saxagliptin and metabolite M2 in the PBPK-DO model.

Property	Saxagliptin	Metabolite M2	Source
	Values	Values	
Molecular weight (M)	315.42 g mol^−1^	331.42 g mol^−1^	Su et al. (2012)
PK^a^	7.3 (base)	7.6 (base)	Obtained from the literature^9^ and Chemspider, respectively
LogP	−1.82 (@pH1.2)	−1.44	Obtained from the literature^9^ and Chemspider, respectively
Effective permeability in humans (P_eff_)	19 × 10^–5^ cm s^−1^	19 × 10^–5^ cm s^−1^	Calculated by ADMET Predictor 7.0
Fraction of free drug (f_up_)	0.95	0.95	Plasma protein binding of both compounds was very low^14^ and hence assigned at 5%
Blood-to-plasma concentration ratio (Rbp)	0.83	0.83	Calculated by ADMET Predictor 7.0
Initial absorption rate constant (K_a,i0_/K_a,met0_)	1.51 h^−1^ (duodenum)	0.25	Calculated based on Eq. 4 for saxagliptin; optimized by PK curves of M2 for the metabolite
	2.25 h^−1^ (jejunum)		
	2.46 h^−1^ (ileum)		
Absorption adjustment factor (f_a_/f_a,met_)	0.005 μg^−1^	0.00015 μg^−1^	Optimized by matching observed T_max_
Scale factor of metabolite conversion (f_scale_)	_	2	Adjusted based on the PK profile of M2 in humans
V_max_ for 3A4	V_max_ = 31.7 pmol M2/pmol CYP/min	_	Su et al. (2012)
K_m_ for 3A4	K_m_ = 81.7 μM	_	
Free drug concentration in the enterocyte compartment (f_ugut_)	1.0	1.0	Defaulted according to the literature Qian et al. (2019)
Intestine/blood concentration ratio (K_p,ent_)	1.03	_	Estimated by Rodgers’ model Rodgers et al. (2005); Rodgers and Rowland (2006)
Liver-to-plasma partition coefficient (K_p,li_)	1.08	1.21	
Kidney-to-plasma partition coefficient (K_p,ki_)	0.74	0.74	
Lung-to-plasma partition coefficient (K_p,lu_)	0.76	0.62	
Non-eliminating-to-plasma partition coefficient (K_p,nt_)	7.8	7.8	Optimized by the observed PK profile
Kidney clearance (CLr)	10.8 L/h	4.6 L/h	Upreti et al. (2011)
On-rate (k_on_) to DPP-4	565.7 μM^−1^h^−1^	2,582.6 μM^−1^h^−1^	Obtained from the literature Kim et al. (2006) for saxagliptin, calculated with k_off_/K_i_ for M2 (K_i_ = 0.7 μM)
Off-rate (k_off_) from DPP-4	0.2 h^−1^	1.8 h^−1^	Obtained from the literature Kim et al. (2006) for saxagliptin and^9^ for M2

### Development of the PBPK-DO Model for Saxagliptin and Metabolite M2

**FIGURE 2:**
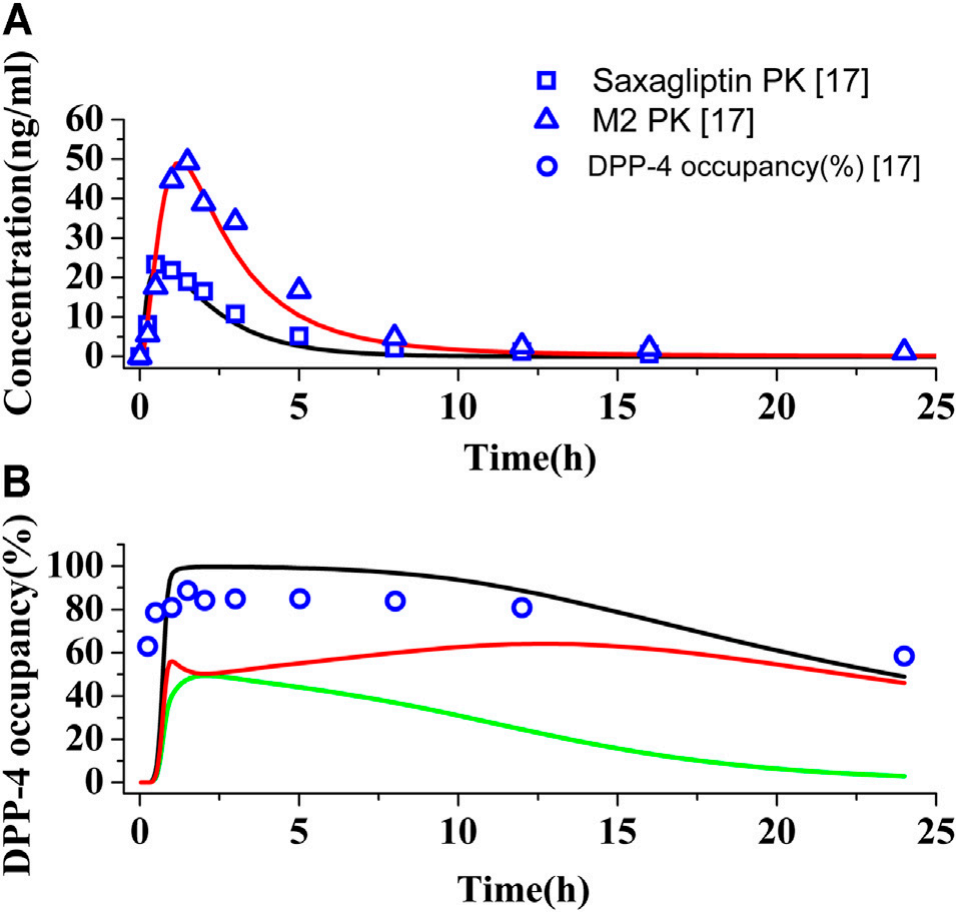
Simulations of pharmacokinetics and DPP-4 occupancy in humans. **(A)** Predicted and observed human plasma concentration–time curves of saxagliptin and M2 after oral administration at a dose of 5 mg. Black (∩) and red (∩) solid lines represent plasma concentration–time curves of saxagliptin and M2, respectively. The blue squares (.) and blue up-triangles (△) refer to experimentally measured pharmacokinetic data of saxagliptin and M2, respectively. **(B)** Predicted and observed time course of DPP-4 occupancy by saxagliptin in humans following oral administration of 5 mg. Black (∩), green (∩), and red (∩) solid lines represent human DPP-4 occupancy curves of saxagliptin + M2, saxagliptin, and M2, respectively. The blue circles (◯) refer to experimentally measured DPP-4 occupancy data.

The PBPK-DO model for saxagliptin has been established by a massive number of input parameters from Table 1 and Supplementary Table S4. Figures 2A,B show the predictions and observations of the human PK and DPP-4 occupancy for saxagliptin and metabolite M2, respectively, after oral administration of a 5 mg saxagliptin. Comparison of observed PK and DPP-4 occupancy parameters with simulated parameters of saxagliptin and M2 is summarized in Supplementary Table S5. It is clearly indicated that human PK simulation corresponds closely to observed values for saxagliptin and M2 (Upreti et al., 2011) and that simulation of time course of DPP-4 occupancy in humans by saxagliptin could also be matched with experimentally determined values very well (Upreti et al., 2011). The simulation results have displayed that the developed PBPK-DO model could accurately predict PK profiles and DPP-4 time profiles in humans for saxagliptin and M2.

### Effect of Inhibitors/Inducers on PK and PD of Saxagliptin in Humans

The PK profile predictions of inhibitors/inducers have been shown in Supplementary Figure S1, and the predicted and observed data are listed in Supplementary Table S6. The accuracies of PK prediction using the developed PBPK model have been verified by comparing predicted and observed PK of the inhibitors/inducers. The comparison displayed that predicted PK profiles matched observed profiles well (Supplementary Figure S1) and that all fold errors were less than 2 between predicted and observed PK data (Supplementary Table S6). The result indicated that the established PBPK model could accurately simulate the PK process in humans of inhibitors and inducers.

**FIGURE 3:**
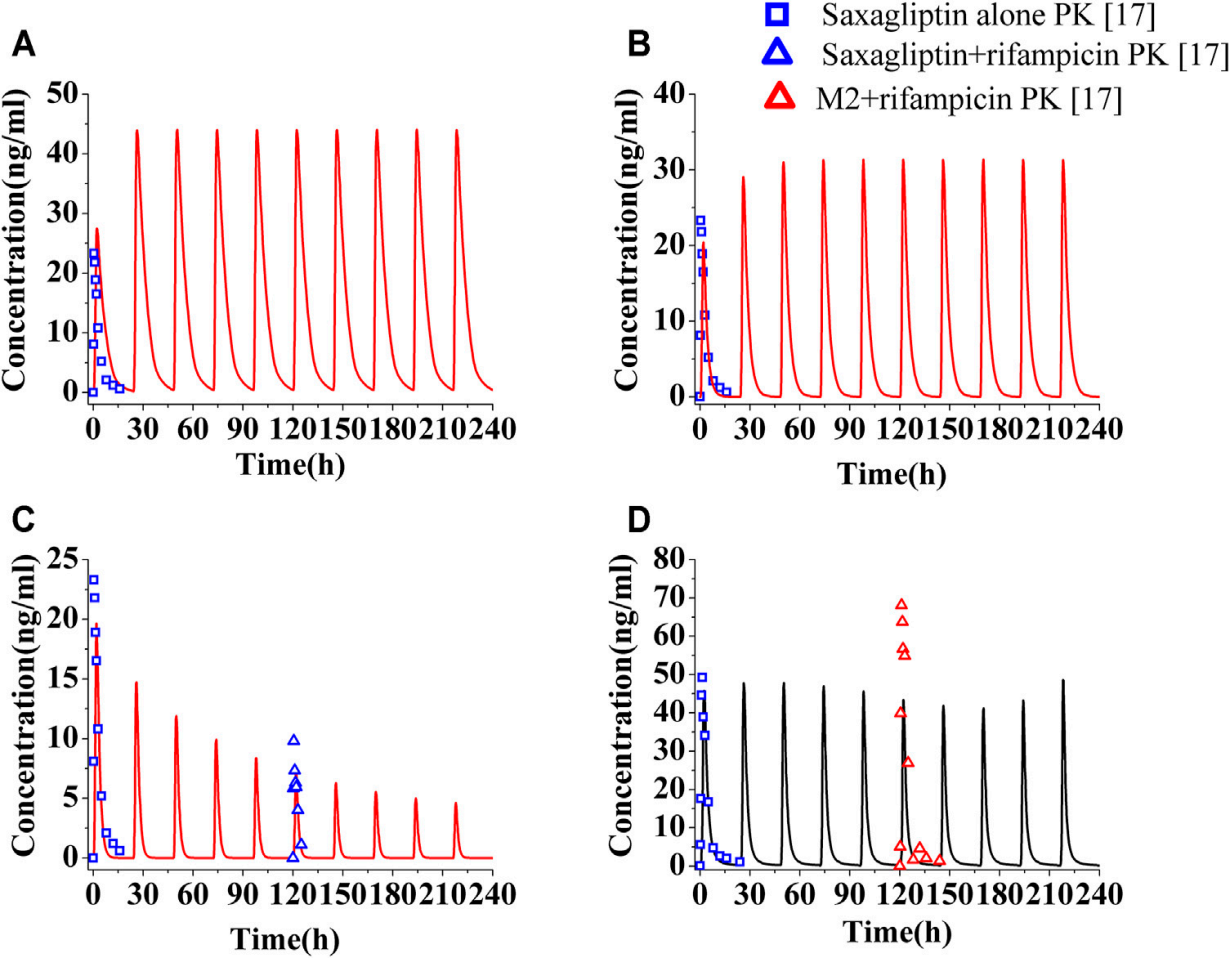
Human plasma level of saxagliptin and M2 following coadministration of inhibitors or inducers. Human plasma profiles of saxagliptin with ketoconazole (**A**, 200°mg, twice daily), with delavirdine (**B**, 400°mg, twice daily) and rifampicin (**C**, 600°mg, once daily) and of M2 with rifampicin **(D)**. The blue squares, blue up-triangles, and red up-triangles refer to observed PK data of saxagliptin without inhibitors/inducers (□) and observed PK data of saxagliptin (△) and M2 (△) with rifampicin on the sixth day, respectively.

**FIGURE 4:**
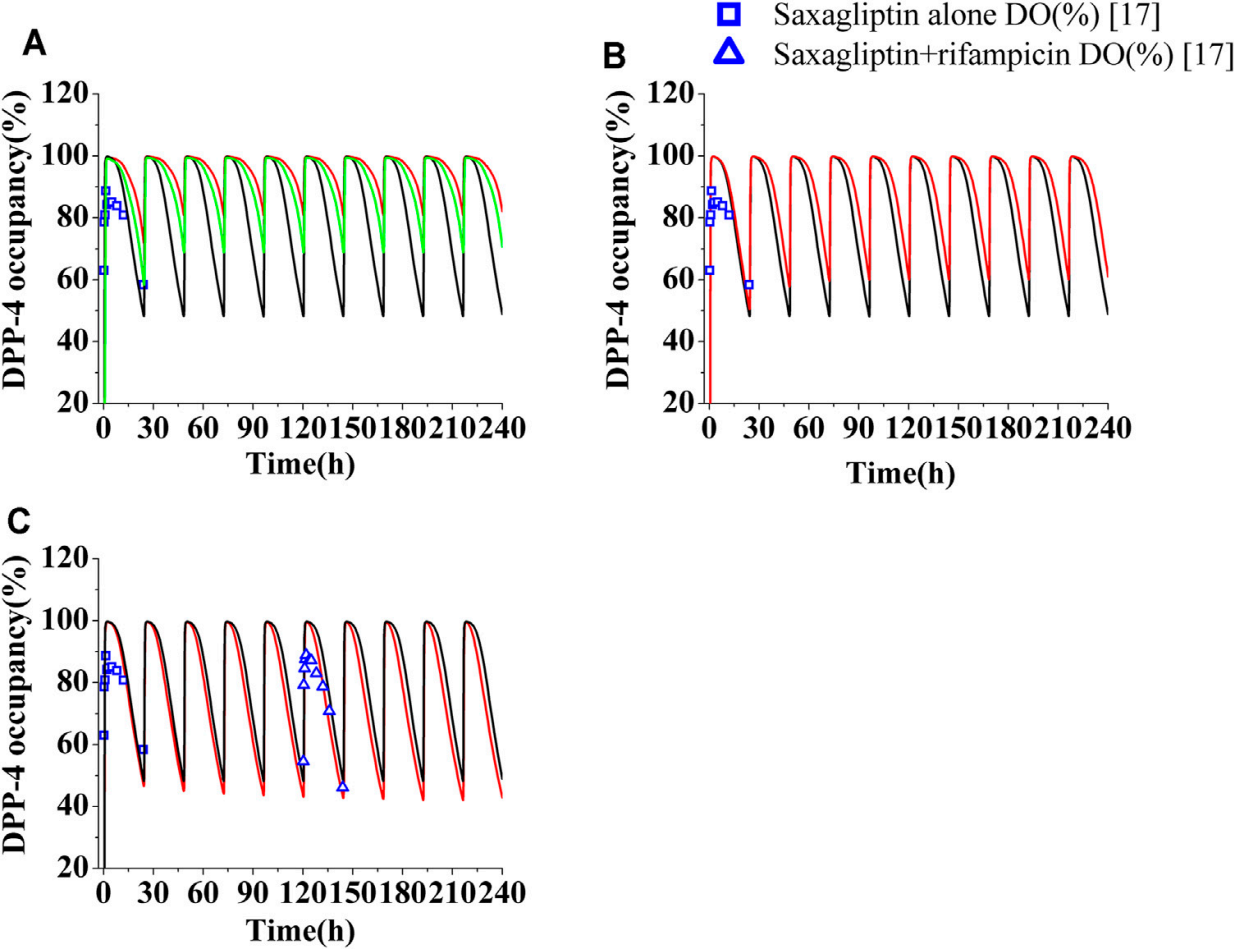
Human DPP-4 occupancy by saxagliptin and M2 following coadministration of inhibitors or inducers. **(A)** Human DPP-4 by saxagliptin of 5 mg (∩) and 2.5 mg (∩) with ketoconazole (200 mg, twice daily). **(B)** Human DPP-4 by saxagliptin of 5 mg (∩) with delavirdine (400°mg, twice daily). **(C)** Human DPP-4 by saxagliptin of 5 mg (∩) with rifampicin (600°mg, once daily). The black lines (∩) represent human DPP-4 by saxagliptin of 5 mg without inhibitors/inducers. The blue squares (□) and blue up-triangles (△) refer to observed DPP-4 occupancy data of saxagliptin and M2 without inhibitors/inducers and observed DPP-4 occupancy data of saxagliptin and M2 with rifampicin on the sixth day, respectively.

The PK profiles and DPP-4 occupancy profiles of saxagliptin in humans at a dose of 5 mg were simulated using the developed PBPK-DO model after coadministration of ketoconazole (200°mg, twice daily), delavirdine (400°mg, twice daily), and rifampicin (600°mg, once daily) for 10°days, respectively (Figures 3, 4). The C_max_ and AUC_0-t_ of saxagliptin coadministrated with multidose ketoconazole on the sixth day increased by 1.89- and 3.42-fold of those without ketoconazole, respectively, (Supplementary Table S7). Slight underestimation was found in C_max_ and AUC of saxagliptin for DDI predictions (C_max_ ratio: 1.89; AUC_0-t_ ratio: 3.42) versus DDI observations (C_max_ ratio: 2.44; AUC_0-t_ ratio: 3.67) after coadministration of ketoconazole^7^. It was observed that C_max_ and AUC of saxagliptin increased by 1.33- and 1.50-fold with delavirdine compared to those without the inhibitor, respectively (Supplementary Table S8). The PK profiles and parameters of saxagliptin were strongly influenced following coadministration of rifampicin, while PK profiles and parameters of metabolite M2 were nearly unchanged (Figure 3D and Supplementary Table S9). Slight overestimation was observed in C_max_ and AUC of saxagliptin compared to actually experimentally determined values, with DDI predictions of a C_max_ ratio of 0.31 and an AUC_0-t_ ratio of 0.19 versus DDI observations of a C_max_ ratio of 0.42 and an AUC_0-t_ ratio of 0.24 (Upreti et al., 2011). The predicted AUC_0-t_ ratio of M2 was slightly below clinical experiment data (0.78 versus 0.91) (Upreti et al., 2011), while the C_max_ ratio of M2 had medium differences between predicted and experimentally determined values (0.88 versus 1.38) (Upreti et al., 2011).

The levels of DPP-4 occupancy by saxagliptin (5 mg) and M2 coadministrated with multidose ketoconazole have been significantly enhanced compared to that without ketoconazole, with the lowest DPP-4 occupancy being >80% (Figure 4A, red line). Owing to significant improvement of DPP-4 occupancy, next, the time course of DPP-4 occupancy was simulated at a lower dose of 2.5 mg (Figure 4A, green line). The comparison between the DPP-4 occupancy profile at 5 mg of saxagliptin without ketoconazole (black line in Figure 4A) and that at 2.5 mg of saxagliptin with ketoconazole (green line in Figure 4A) demonstrated that oral saxagliptin should be decreased to 2.5 mg when there is coadministration with ketoconazole. The result was in good agreement with reported data in the literature^7^. Although AUC of saxagliptin increased 1.5-fold with delavirdine, however, the DPP-4 occupancy time profile by saxagliptin and M2 coadministrated with multidose delavirdine almost coincided with that without delavirdine (Figure 4B). In contrast, while C_max_ and AUC of saxagliptin with coadministration of rifampicin had a considerable decrease, the percent occupancy of DPP-4 by saxagliptin and M2 with coadministration of rifampicin closely resembled that without rifampicin (Figure 4C). This prediction was in line with the published result in the study (Upreti et al., 2011). The similar occupancy of DPP-4 indicated that M2 was likely the main contributor to human DPP-4 occupancy under the circumstance of coadministration with rifampicin.

## Discussion

In this study, we have developed a PBPK-DO mathematical model characterized by two simultaneous simulations (parent/metabolite and PK/PD), which was first utilized to quantify the impacts of CYP3A4 inhibitors and inducers on the PK and PD of saxagliptin and M2 in humans simultaneously. Ketoconazole and rifampicin are recommended as the standard CYP3A4 competitive inhibitor and inducer for the potential clinical DDI study, respectively. In addition, we also assessed the effect of a TDI (delavirdine) on the PK and PD of saxagliptin and M2, which is a distinct inhibition type from ketoconazole.

Although some studies have showed that P450 metabolic activity of a drug could be frequently different in the gut and the liver (Choe et al., 2017; Kapetas et al., 1208; Kurucz et al., 2019; Klomp et al., 2020), the metabolic parameter of saxagliptin by intestinal CYP3A4 was considered comparable to hepatic CYP3A4 in this PBPK-DO model. f_a_ and ASF were incorporated into this model to optimize PK peak time of saxagliptin and scale the effective permeability^4,^ ^6^, which greatly improved prediction performance. According to the literature (Upreti et al., 2011; Boulton, 2017), here, we assumed that all DPP-4 enzymes were located in the blood compartment in the present model. Across published studies (Rowland Yeo et al., 2011; Bolleddula et al., 2021), k_deg_ values of CYP3A4 ranged from 0.0077^–1^ to 0.03 h^−1^. However, a recent study has confirmed that it was a more reasonable value at 0.03 h^−1^ for the most accurate prediction (Rowland Yeo et al., 2011); hence, the k_deg_ value was set at 0.03 h^−1^ in this PBPK-DO model. The induction activity (E_max_ and EC_50_) of rifampicin suggested major individual variability between different literature studies (Yamazaki et al., 2015; Almond et al., 2016; Asaumi et al., 2018; Hanke et al., 2018). To minimize the variation, we used mean values of E_max_ (6.2 μm) and EC_50_ (0.6 μm) in the current model (Qian et al., 2019). Use of rifampicin induction parameters could have been rationalized indirectly that the predicted expression amount of mean intestinal CYP3A4 after induction by rifampicin was close to the experimentally determined value in humans^8^ (6.4-fold change versus 4.4-fold change). The content of human microsomal protein was set at 38 mg/g in the liver^6^ rather than 45 mg/g liver in some studies (Guo et al., 2013; Qian et al., 2019) because the former parameter had been proved to be a more reasonable value in more studies and built-in business software^6^.

All predictions were within 2-fold of observations among them, and the highest fold error was 1.5-fold, which occurred between the predicted and observed C_max_ ratios of M2 with rifampicin. In accordance with FDA clinical DDI guidance (Sudsakorn et al., 2020), if the AUC ratio of a drug with and without the inhibitor is ≥1.25 or ≤0.8 with and without the inducer, clinically relevant DDI should be considered. In our simulations, the AUC ratio of saxagliptin was found to be 1.50 with delavirdine and to be 0.19 with rifampicin, which could occur in significant clinical DDIs based predominantly on PK comparisons. Nevertheless, DPP-4 occupancy by saxagliptin with delavirdine or rifampicin was almost unchanged, and PD simulations displayed that delavirdine or rifampicin would not cause human DDIs for saxagliptin, which was in good agreement in clinical experiments (Upreti et al., 2011). The simulation results also have further demonstrated the importance of two simultaneous simulations in the present model.

It was reported that the C_max_ and AUC_0-t_ of saxagliptin increased by less than 2-fold in patients with severe hepatic impairment but by more than 2-fold in patients with severe renal impairment, respectively (Boulton, 2017). PK simulation in humans with hepatic or renal impairment is performed by modulating many physiological parameters based on healthy humans (Malik et al., 2020). However, currently, this simulation model cannot simulate PK of patients with hepatic or renal impairment yet. Recent literature reported that catalytic activities of 27 CYP3A4 variants on the *in vitro* metabolism of saxagliptin were evaluated (Liu et al., 2021). CYP3A4 variants showed decreased activities ranging from 1.9 to 77.1% as compared to the wild type. Hence, we also preliminarily evaluated the effect of genetic variations in metabolizing enzymes on PK and PD of saxagliptin using this model. Here, we only simulated the PK and PD of saxagliptin in humans with variant CYP3A4*22. In the simulation, expression and activity of CYP3A4 were replaced with 59 and 40% of the wild type (Alqahtani and Kaddoumi, 2016). The results displayed that C_max_ and AUC_0-t_ of saxagliptin increased by about 2-fold, and DPP-4 occupancies by saxagliptin between three oral doses (5, 2.5, and 1 mg) have a slight difference (Supplementary Table S10 and Supplementary Figure S2). Of note, due to clinical data unavailability, prediction accuracy need be proven with further *in vivo* studies.

## Conclusion

Taken together, this mathematic model is characterized by two simultaneous simulations (parent/metabolite and PK/PD), describing two interaction processes between inhibitors/inducer-CYP3A4 and saxagliptin/M2-DPP-4. We conceive that compared to most current single PK-DDI predictions, the wide application of the PBPK-DO model has the power to improve the predictions of potential clinical DDIs for victim drugs metabolized by CYP3A4.

## Data Availability

The original contributions presented in the study are included in the article/Supplementary Material further inquiries can be directed to the corresponding authors.

## Footnotes

1 Saxagliptin.pdf

2 Clinical Pharmacokinetics of Ketoconazole.pdf

3 Delavirdine mesylate and didanosine

4 PBPK book.pdf

6 GastroPlusManual-9.7.pdf

7 Saxagliptin instruction.pdf

8 ClinPharmR_P1.pdf

9 The role of intestinal P-glycoprotein.pdf

14 IF.pdf
