# Supplementary material for: Effect of CYP3A4 Inhibitors and Inducers on Pharmacokinetics and Pharmacodynamics of Saxagliptin and Active Metabolite M2 in Humans Using Physiological-Based Pharmacokinetic Combined DPP-4 Occupancy
Source: Front Pharmacol. 2021 Oct 19;12:746594. doi: 10.3389/fphar.2021.746594 (PMC8560969; doi:10.3389/fphar.2021.746594)
Supplement: Supplementary file 1 [file DataSheet1.docx]

Supplementary Material

# Supplementary Figures and Tables

## Supplementary Figures





**Supplementary Figure S1 The predicted and observed human plasma concentration-time curves of inhibitors and inducer.** Predicted and observed human plasma concentration-time curves of ketoconazole (A), delavirdine (B) and rifampicin (C) after oral administration of 400 mg, 400 mg and 600 mg, respectively. The blue squares (🞏) refer to experimentally measured pharmacokinetic data of inhibitors and inducer.





**Supplementary Figure S2 The predicted DPP-4 occupancy-time curves in human with variant CYP3A4*22of saxagliptin at three oral doses.**

## Supplementary Tables

| **Supplementary Table S1** Summary of input parameters for ketoconazole in PBPK model | | |
| --- | --- | --- |
| Property | Values | Source |
| Molecular weight (M) | 531.4 g·mol-1 | Chemspider |
| pKa | 6.51 (base) | Obtained from literature[32] |
| LogP | 2.67 (@pH7.4) | Calculated by ADMET Predictor 7.0 |
| Effective permeability in human (P_eff_) | 1.24🞨10^-5^ cm⋅s^-1^ | Calculated by ADMET Predictor 7.0 |
| Fraction of free drug (f_up_) | 0.015 | Obtained from literature[33] |
| Blood-to-plasma concentration ratio (Rbp) | 0.59 | Obtained from literature[34] |
| Initial absorption rate constant (K_a,i0_) | 0.1 h^-1^ (duodenum)  0.15 h^-1^ (jejunum)  0.16 h^-1^ (ileum) | Calculated based on equation (4) |
| Absorption adjustment factor (f_a_) | 0.0003 | Optimized by matching observed T_max_ |
| V_max_ for 3A4 | V_max_（gut）=10 μg/mg CYP/min  V_max_（liver）=0.0289μg/mg CYP/min | Built-in libraries |
| K_m_ for 3A4 | K_m_=8μg/L |  |
| Free drug concentration in enterocyte compartment (f_ugut_) | 1.0 | Defaulted according to literature[18] |
| Intestine/blood concentration ratio (K_p,ent_) | 0.42 | Estimated by Rodgers’ model[29,30] |
| Liver-to-plasma partition coefficient (K_p,li_) | 0.23 |  |
| Kidney-to-plasma partition coefficient (K_p,ki_) | 0.26 |  |
| Lung-to-plasma partition coefficient (K_p,lu_) | 0.34 |  |
| Non-eliminating-to-plasma partition coefficient (K_p,nt_) | 3.0 | Optimized by observed PK profile |
| Kidney clearance（CLr） | 0.11 L/h | Estimated based on fup*GFR method, defaulted GFR for adults is~120 mL/min |
| Inhibition constant against CYP3A (K_i_) | 0.015 μM | Obtained from literature [35] |

| **Supplementary Table S2** Summary of input parameters for delavirdine in PBPK model | | |
| --- | --- | --- |
| Property | Values | Source |
| Molecular weight (M) | 456.57 g·mol-1 | Chemspider |
| pKa | 9.39 (acid); 4.56 (base) | Calculated by ADMET Predictor 7.0 |
| LogP | 1.02 | Drugbank |
| Effective permeability in human (P_eff_) | 9.1🞨10^-5^ cm⋅s^-1^ | Calculated by ADMET Predictor 7.0 |
| Fraction of free drug (f_up_) | 0.02 | Obtained from literature[36] |
| Blood-to-plasma concentration ratio (Rbp) | 0.87 | Calculated by ADMET Predictor 7.0 |
| Initial absorption rate constant (K_a,i0_) | 0.73 h^-1^ (duodenum)  1.07 h^-1^ (jejunum)  1.18 h^-1^ (ileum) | Calculated based on equation (4) |
| Absorption adjustment factor (f_a_) | 0.000005 | Optimized by matching observed T_max_ |
| V_max_ for 3A4 | V_max_=440 pmol/mg CYP/min | Obtained from literature[37] |
| K_m_ for 3A4 | K_m_=6.8μM |  |
| Free drug concentration in enterocyte compartment (f_ugut_) | 1.0 | Defaulted according to literature[18] |
| Intestine/blood concentration ratio (K_p,ent_) | 0.18 | Estimated by Rodgers’ model[29,30] |
| Liver-to-plasma partition coefficient (K_p,li_) | 0.10 |  |
| Kidney-to-plasma partition coefficient (K_p,ki_) | 0.15 |  |
| Lung-to-plasma partition coefficient (K_p,lu_) | 0.19 |  |
| Non-eliminating-to-plasma partition coefficient (K_p,nt_) | 2.0 | Optimized by observed PK profile |
| Kidney clearance（CLr） | 0.14 L/h | Estimated based on fup*GFR method, defaulted GFR for adults is~120 mL/min |
| The maximum rate of inactivation against CYP3A (k_inact_) | 0.056 min^-1^ | Obtained from literature [38] |
| Inhibition constant against CYP3A (K_i_) | 5.2 μM |  |

| **Supplementary Table S3** Summary of input parameters for rifampicin in PBPK model | | |
| --- | --- | --- |
| Property | Values | Source |
| Molecular weight (M) | 822.94 g·mol-1 | Chemspider |
| pKa | 1.7 (acid); 7.9 (base) | Obtained from literature[39] |
| LogP | 3.85 | Obtained from literature[40] |
| Effective permeability in human (P_eff_) | 4.0🞨10^-5^ cm⋅s^-1^ | Obtained from literature[18] |
| Fraction of free drug (f_up_) | 0.17 | Obtained from literature[41] |
| Blood-to-plasma concentration ratio (Rbp) | 0.89 |  |
| Initial absorption rate constant (K_a,i0_) | 0.31 h^-1^ (duodenum)  0.48 h^-1^ (jejunum)  0.52 h^-1^ (ileum) | Calculated based on equation (4) |
| Absorption adjustment factor (f_a_) | 0.000003 | Optimized by matching observed T_max_ |
| Free drug concentration in enterocyte compartment (f_ugut_) | 1.0 | Defaulted according to literature[18] |
| Intestine/blood concentration ratio (K_p,ent_) | 12.3 | Estimated by Rodgers’ model[29,30] |
| Liver-to-plasma partition coefficient (K_p,li_) | 6.3 |  |
| Kidney-to-plasma partition coefficient (K_p,ki_) | 5.76 |  |
| Lung-to-plasma partition coefficient (K_p,lu_) | 7.65 |  |
| Non-eliminating-to-plasma partition coefficient (K_p,nt_) | 0.3 | Optimized by observed PK profile |
| Liver intrinsic clearance (CLli) | 14.8 L/h | Obtained by unit conversion from the literature [18] |
| Kidney clearance（CLr） | 0.92 L/h | Obtained from literature [18] |
| Maximum inductive effect for CYP3A (E_max_) | 6.2 μM | Obtained from literature [18] |
| Inducer concentration required to achieve 50% inductive effect (EC_50_) | 0.6 μM | Obtained from literature [18] |

**Supplementary Table S4 Physiological property for the PBPK-DO model in human**

| Property | Values | Source |
| --- | --- | --- |
| Gastric emptying rate (K_0_) | 2.11 h^-1^ | 18 |
| Gut transit rate constant (K_t,i_) | 4.07 h^-1^ (duodenum)  1.62 h^-1^ (jejunum)  2.19 h^-1^ (ileum) |  |
| Mean radius of gut lumen (r_i_) | 2.0 cm(duodenum)  1.63 cm (jejunum)  1.45 cm (ileum) |  |
| Mean absorption scale factor (ASF) | 2.23 (duodenum)  2.68 (jejunum)  2.62 (ileum) | Built-in libraries |
| Enterocytic blood flow (Q_ent,i_) | 2.7 L/h (duodenum wall)  10.4 L/h (jejunum wall)  6.1 L/h (ileum wall) | 18 |
| Enterocytic volume (V_ent,i_) | 0.021 L (duodenum wall)  0.063 L (jejunum wall)  0.042 L (ileum wall) |  |
| Portal vein blood flow (Q_pv_) | 53 L/h | 22 |
| Portal vein volume (V_pv_) | 0.07 L |  |
| Liver blood flow (Q_li_) | 78.1 L/h |  |
| Hepatic artery blood flow(Q_lia_) | 25.1 L/h |  |
| Liver volume (V_li_) | 1.34 L |  |
| Kidney blood flow (Q_ki_) | 53.3 L/h |  |
| Kidney volume (V_ki_) | 0.227 L |  |
| Lung blood flow (Q_lu_) | 310.3 L/h |  |
| Lung volume (V_lu_) | 0.194 L |  |
| blood flow sum of non-eliminating tissue (Q_nt_) | 189 L/h |  |
| Mean volume of non-eliminating tissue (V_nt_) | 5.9 L |  |
| Arterial blood volume (V_ab_) | 1.78 L |  |
| Venous blood volume (V_vb_) | 3.56 L |  |
| Microsomal protein | 38 mg/g liver | Built-in libraries |
| Enterocytic CYP3A abundance(Abundance_ent,i_) | 9.7 nmol (duodenum wall)  38.4 nmol (jejunum wall)  22.4 nmol (ileum wall) | 18 |
| Hepatic CYP3A abundance(Abundance_li_) | 9712.8 nmol | 20 |
| Degradation rate constant of CYP3A (k_deg_) | 0.03 h^-1^ | Built-in libraries |

**Supplementary Table S5 The observed and predicted PK and PD for saxagliptin and M2 according to PBPK-TO model**

| Drug | Parameters | Observed | Predicted | Fold error ^a^ |
| --- | --- | --- | --- | --- |
| Saxagliptin | C_max_ (ng·mL^-1^) | 23.3 | 22.5 | 1.04 |
|  | AUC_0-inf_ (ng·h·mL^-1^) | 91.36 | 63.21 | 1.45 |
|  | AUC_0-16_ (ng·h·mL^-1^) | 87.23 | 63.19 | 1.38 |
| Metabolite M2 | C_max_ (ng·mL^-1^) | 49.2 | 49.1 | 1.00 |
|  | AUC_0-inf_ (ng·h·mL^-1^) | 209.01 | 172.41 | 1.21 |
|  | AUC_0-24_ (ng·h·mL^-1^) | 207.75 | 170.11 | 1.22 |
| Saxagliptin+ M2 | TO_max_ (%) | 88.7 | 99.7 | 1.12 |
|  | TO_AUC(0-24)_(%·h) | 1806.2 | 1847.4 | 1.02 |
|  | D_TO >60%_ (h) | ≈24 | 18.8 | 1.28^b^ |

^a^Calculated using observed/predicted, if observed value is less than the predicted value, fold error=predicted/observed

^b^Observed value is defaulted to be 24 when calculated

**Supplementary Table S6 The observed and predicted PK for the inhibitors/inducer according to PBPK model**

| Drug | Parameters | Observed | Predicted | Fold error |
| --- | --- | --- | --- | --- |
| Ketoconazole | C_max_ (ng·mL^-1^) | 6240 | 6574.4 | 1.05 |
|  | AUC_0-inf_ (ng·h·mL^-1^) | 43637.7 | 51052.0 | 1.17 |
|  | AUC_0-48_ (ng·h·mL^-1^) | 43017.1 | 49884.3 | 1.16 |
| Delavirdine | C_max_ (ng·mL^-1^) | 3957.8 | 4186.5 | 1.06 |
|  | AUC_0-inf_ (ng·h·mL^-1^) | 20342.4 | 19018.5 | 1.07 |
|  | AUC_0-12_ (ng·h·mL^-1^) | 18612.6 | 18623.3 | 1.00 |
| Rifampicin | C_max_ (ng·mL^-1^) | 9540 | 10068 | 1.06 |
|  | AUC_0-inf_ (ng·h·mL^-1^) | 66401.6 | 71284.3 | 1.07 |
|  | AUC_0-24_ (ng·h·mL^-1^) | 64897.9 | 70089.9 | 1.08 |

**Supplementary Table S7 DDI parameter changes of saxagliptin and M2 with or without ketoconazole**

| Drug | Parameters | Observed without  ketoconazole | Predicted with ketoconazole | Predicttion-to-observation ratio |
| --- | --- | --- | --- | --- |
| Saxagliptin | C_max_ (ng·mL^-1^) | 23.3 | 44.0 | 1.89 |
|  | AUC_0-inf_ (ng·h·mL^-1^) | 91.36 | 344.2 | 3.77 |
|  | AUC_0-t_ (ng·h·mL^-1^) | 87.23 | 298.0 | 3.42 |
| Saxagliptin+M2（5mg） | TO_max_ (%) | 88.7 | 99.7 | 1.12 |
|  | D_TO >60%_ (h) | ≈24 | >24 | - |
| Saxagliptin+M2（2.5mg） | TO_max_ (%) | 88.7 | 99.4 | 1.12 |
|  | D_TO >60%_ (h) | ≈24 | >24 | - |

**Supplementary Table S8 DDI parameter changes of saxagliptin and M2 with or without delavirdine**

| Drug | Parameters | Observed  without delavirdine | Predicted with delavirdine | Predicttion-to-observation ratio |
| --- | --- | --- | --- | --- |
| Saxagliptin | C_max_ (ng·mL^-1^) | 23.3 | 31.3 | 1.34 |
|  | AUC_0-inf_ (ng·h·mL^-1^) | 91.36 | 131.6 | 1.44 |
|  | AUC_0-t_ (ng·h·mL^-1^) | 87.23 | 130.6 | 1.50 |
| Saxagliptin+M2（5mg） | TO_max_ (%) | 88.7 | 99.7 | 1.12 |
|  | D_TO >60%_ (h) | ≈24 | 22.1 | - |

**Supplementary Table S9 DDI parameter changes of saxagliptin and M2 with or without rifampicin**

| Drug | Parameters | Observed without  rifampicin | Predicted with rifampicin | Predicttion-to-observation ratio |
| --- | --- | --- | --- | --- |
| Saxagliptin | C_max_ (ng·mL^-1^) | 23.3 | 7.2 | 0.31 |
|  | AUC_0-inf_ (ng·h·mL^-1^) | 91.36 | 16.8 | 0.18 |
|  | AUC_0-t_ (ng·h·mL^-1^) | 87.23 | 16.8 | 0.19 |
| Metabolite M2 | C_max_ (ng·mL^-1^) | 49.2 | 43.3 | 0.88 |
|  | AUC_0-inf_ (ng·h·mL^-1^) | 209.01 | 174.3 | 0.83 |
|  | AUC_0-24_ (ng·h·mL^-1^) | 207.75 | 163.0 | 0.78 |
| Saxagliptin+M2 | TO_max_ (%) | 88.7 | 99.5 | 1.12 |
|  | D_TO >60%_ (h) | ≈24 | 17 | - |

**Supplementary Table S10 Predicted PK and PD of saxagliptin in human with CYP3A4 variants**

| Drug | Parameters | Predicted |
| --- | --- | --- |
| Saxagliptin（5mg） | C_max_ (ng·mL-1) | 40.8 |
|  | AUC_0-inf_ (ng·h·mL-1) | 183.64 |
|  | AUC_0-t_ (ng·h·mL-1) | 183.54 |
| Saxagliptin+M2（5mg） | TO_max_ (%) | 99.8 |
|  | DTO >60% (h) | >24 |
| Saxagliptin+M2（2.5mg） | TO_max_ (%) | 99.5 |
|  | DTO >60% (h) | 23.6 |
| Saxagliptin+M2（1mg） | TO_max_ (%) | 98.2 |
|  | DTO >60% (h) | 23.5 |
